# Informal Networks, Informal Institutions, and Social Exclusion in the Workplace: Insights from Subsidiaries of Multinational Corporations in Korea

**DOI:** 10.1007/s10551-022-05244-5

**Published:** 2022-09-08

**Authors:** Sven Horak, Yuliani Suseno

**Affiliations:** 1 St. John’s University, The Peter J. Tobin College of Business, Bent Hall, Queens, USA; 2 School of Management, College of Business and Law, RMIT University, Melbourne, VIC 3000 Australia

**Keywords:** Informal networks, Informal institutions, *Yongo*, Social exclusion, Gender, Women’s careers

## Abstract

Drawing on interviews with decision makers in multinational corporations (MNCs) in South Korea, we examine the role of informal networks in the social exclusion of women in the workforce. Although legislation in the country is in favor of gender equality, we found that informal barriers in the workplace remain difficult to overcome. Informal networks in Korea, *yongo*, present an ethical issue in the workplace, as they tend to socially exclude women, limiting possibilities for their participation and career progression. We found that informal networks are pervasive and strong because of the informal institutions in which they are embedded and that there is a complex interplay between informal networks and informal institutions that socially excludes women. Due to difficulties accessing *yongo*, women appear to build *inmaek*, a network type that is more open and accessible. We also found that MNCs in Korea can compensate for the lack of local informal networks for their female employees. However, despite providing a more supportive environment for women at work, gender equality policies in MNCs are not yet as effective as they could be due to the dynamics of the workplace and the fact that the policies are not tailored to the local context. We have seen evidence in recent years that MNCs can serve as role models for implementing gender equality policies by creating a more inclusive work environment and demonstrating leadership commitment and support.

## Introduction

While previous studies highlighted the importance of networks in business relationships and exchanges (Cuypers et al., 2020; Eberhard & Craig, 2013), research on informal networks in particular remains limited, and the topic warrants further investigation (Georgiadou & Syed, 2021; Minbaeva et al., 2022). Informal networks, which rely on personal ties and connections in interactions with others, can play a role in enabling certain practices and facilitating business relationships. For example, Nadeem and Kayani (2019) illustrated how informal social ties are a cornerstone of traditional values in Pakistan and how they influence hiring practices in the country. Apaydin et al. (2020) highlighted that an informal network of local merchants could be a crucial intermediary in the internationalization process of multinational enterprises. These informal networks are anchored in a society’s social fabric as well as its cultural and societal traditions, which evolve over time (Horak, 2014). As a result, informal networks channel continuity and change in informal institutions (Minbaeva et al., 2022), potentially posing challenges to individuals and organizations (Horak et al., 2020; Nadeem & Kayani, 2019).

Existing studies on informal networks primarily focused on Western societies’ notions and knowledge of such networks, necessitating a deeper understanding of “local phenomena in order to draw a more realistic picture of the true characteristics and nature of social networks” (Horak et al., 2019, p. 368). Informal networks place a strong emphasis on cooperation, trust, and mutuality, as well as an implicit understanding of shared values, norms, and expectations. However, access to these informal networks is not equally available to all individuals (Bian et al., 2015, 2018; Horak & Klein, 2016). Stereotypical gender norms and expectations exist in various societies. As a result, different expectations and norms are imposed on groups of individuals, including women and minorities.

Studies have shown the significance of informal networks, particularly in countries with underdeveloped or absent formal institutions to support market transactions, such as governments and legal/regulatory bodies (Peng et al., 2009; Puffer et al., 2010). However, informal networks persist, even in nations with robust legal and regulatory structures (Horak & Klein, 2016; Minbaeva et al., 2022). The persistence of informal institutions can be explained by the informal networks in which these institutions are embedded (Minbaeva et al., 2022). Therefore, in this paper we focus on the interplay between informal networks (Horak et al., 2020; Lee et al., 2022; Minbaeva et al., 2022) and informal institutions (cultural/societal norms and expectations) (North, 1990, 1991; Williamson, 1998, 2000). Drawing on recent studies that advocated for the further examination of informal networks and informal institutions (Horak et al., 2020; Minbaeva et al., 2022), we specifically examine the interplay between them, focusing on how this interaction leads to the social exclusion of women (Georgiadou & Syed, 2021).

The issue of social exclusion of women is an area in the realm of business ethics concerning diversity and inclusion that has rarely been explored (Georgiadou & Syed, 2021). Societal norms may contribute to the perpetuation of gender inequality in societies with rigid gender stereotypes, where males often occupy the highest positions in corporate hierarchies. As a result of these norms, women find it difficult to network with people in positions of power and influence (Greguletz et al., 2019). Women are also frequently excluded from business conversations because they are not part of the informal networks, thus, denying them access to critical knowledge, resources, and opportunities (Georgiadou & Syed, 2021). As a result, gender segregation and discrimination are still prevalent in the workplace and in society, even in the presence of strong formal institutions.

The interaction between informal networks and informal institutions is context-dependent; therefore, it is crucial to examine this within a social setting. We use the unique workplace setting of South Korea (hereafter referred to as Korea). Adopting Minbaeva et al.’s (2022) argument, we consider the informal networks, known as *yongo* in Korean (Horak, 2014; Kim, 2000; Lee et al., 2022; Yee, 2000), and the informal institutions of norms and expectations as intertwined; together, they function as an enabler of women’s social exclusion in the workplace. In this regard, while we explore the broader issue of why women are still socially excluded in Korea in the twenty-first century, our study specifically formulates the following research question: “How does the interplay between informal networks and informal institutions lead to the social exclusion of women professionals in the workplace in Korea?” Given the continuing nature of workplace discrimination and the growing concerns about business practices in Korea, the social exclusion of women in the Korean workplace is a problem that merits further examination.

Similar to other studies (e.g., Festing et al., 2015), we position our study in the context of subsidiaries of multinational corporations (MNCs) in Korea by conducting interviews with decision makers in these MNC subsidiaries at different time intervals (2009–2019). Multinational corporations, particularly those operating in developing and emerging nations, frequently need to deal with fragmented institutional systems in host markets, where they must contend with a range of social, political, and cultural concerns and constraints (Saka-Helmhout et al., 2016). Despite the fact that many organizations are committed to addressing issues to ensure gender equality, there is still a lack of empirical evidence regarding how MNC subsidiaries deal with informal networks and informal institutions (Horak, 2018; Horak et al., 2020; Lee et al., 2022) or how they address the processes of social exclusion in a host country (Siegel et al., 2019). By exploring and learning about their views, we are able to assess whether the MNC subsidiaries’ initiatives are likely to address social exclusion. Therefore, building on existing studies on business ethics, diversity, and inclusion in international business, our second research question explores the question: “How can MNCs better implement gender equality policies in countries like Korea, where social exclusion is prevalent?”

This study contributes to the literature in several ways. First, the study adds knowledge to the informal networks literature by exploring the interplay between informal networks and informal institutions (Minbaeva et al., 2022). Knowledge of informal networks is important to understand the power of informal relationships (Henry et al., 2004), yet our knowledge on how informal networks are enacted and influenced by informal institutions is still limited (Horak & Yang, 2016; Lee et al., 2022; Minbaeva et al., 2022).

Second, by examining how informal networks and informal institutions facilitate women’s social exclusion from career progression, our study contributes to the body of literature on business ethics in general and gender diversity and inclusion in particular (Georgiadou & Syed, 2021). As Confucianism has had a significant influence on Korea’s economic growth and successes (Choi & Woo, 2018; Lew, 2013), Confucian ideology in regard to gender and women’s roles is still prevalent (Won, 2015; Won & Pascall, 2004). Exploring the significance of informal networks in the domain of business ethics in this context broadens the discourse on gender discrimination, diversity, and inclusion, particularly as our society as a whole strives for a transformative agenda for gender equality and women’s empowerment (Cornwall & Rivas, 2015).

Third, the study contributes to the international business literature by focusing on understanding MNCs and their policies (Siegel et al., 2019). Specifically, we look at how MNCs can be role models by adopting and implementing gender equality-related policies to reduce or eliminate social exclusion in the countries in which they operate. Gender-based discrimination is an emerging research area in international business (Alsarhan et al., 2021; Koveshnikov et al., 2019; Nyarko, 2022), and research in this area has only slowly progressed to examining gender equality practices in MNCs (e.g., Cho et al., 2019; Festing et al., 2015). The study is relevant to not only scholars but also MNCs, since, as part of their global diversity and inclusion policies, many are committed to an inclusive global corporate culture, with equal opportunities for men and women (Saeed et al., 2021).

## The Context

The context of our research was critical. South Korea has experienced significant improvements in female participation and educational attainment (Kim et al., 2016). Female university students accounted for roughly 45% of all students in Korean higher education in 2020, up from 38.3% in 2000 (Statista, 2020). Despite an increase in the number of female university graduates in Korea, participation in the labor force by Korean women is only increasing slowly (Ma, 2016). This is evidenced by Korea’s ranking in the 2021 Global Gender Gap Report (102 out of 156 countries), with the country being listed between Mexico and Vanuatu in terms of economic participation and opportunity (World Economic Forum, 2021).

Korea also ranked last in the 2021 glass-ceiling index (ranking 29), a position it has held for many years due to the low representation of women on boards of directors, in management, and in parliament (Patterson & Walcutt, 2014; The Economist, 2021). Only 15.6% of senior and managerial positions in Korea are held by women (World Economic Forum, 2021). Korea also ranks poorly in wage equality and gender ratio, particularly among legislators, senior officials, and managers (Kim et al., 2016), with the country being able to close only 18.5% of the gender parity gap to date (World Economic Forum, 2021). This gender parity gap is significant, given that the Korean government enacted a comprehensive package of formal rules and labor legislation decades ago, such as the Equal Employment Act in 1987, aimed at protecting women and enhancing equality in the Korean workplace.

Despite its rapid economic growth, Korea remains a patriarchal society with gendered family roles based on Confucian familism (Lee, 2018; Lee et al., 2016; Tung et al., 2013). Research has found that the entrenched patriarchal structures, favoring men and excluding women from being hired and promoted, can lead to negative economic effects (Alsarhan et al., 2021; Kim, 2008). Gender-balanced economies, on the other hand, fare significantly better in terms of their economic performance (Council on Foreign Relations, 2021; OECD, 2014). Interestingly, discrimination against women persists in Korea (Kim et al., 2016). For example, the former chief executive officer (CEO) of the Korea Gas Safety Corporation was found guilty of altering interview scores to favor and select men over women during the recruitment process (Jung, 2018). Three of the largest Korean banks, KB Kookmin Bank, KEB Hana Bank, and Shinhan Bank, were also found to have manipulated the passing scores of female applicants and to have favored men for jobs (Jeong, 2019). The disparities in income between men and women are further exacerbated by unequal access to the labor market, with Korean women being less likely to be in the workforce after the age of 30 (Finch & Kim, 2017). In the following sections, we argue that the social exclusion of women in the workplace is partly due to the prevalence of informal networks that are underpinned by and entrenched in informal institutions. This interconnection presents several underlying gender discriminatory practices that exclude women, hence, constraining and limiting women’s career advancement.

## Theoretical Background

### Social Exclusion

Within the diversity and inclusion research area, social exclusion became a widely acknowledged issue in the mid-1990s. Some scholars regarded social exclusion as a counteragent to social capital (Daly & Silver, 2008; Wilson, 2006), with social exclusion risking the creation of social capital characterized by norms of reciprocity and trust. The concept of social exclusion is often divided into two forms (Wesselman & Williams, 2017): rejection (Richman et al., 2016) and ostracism (Williams & Nida, 2011; Yang & Treadway, 2018). Individuals experiencing rejection feel that they are not wanted and do not belong (Mendoza-Denton et al., 2002; Wesselman et al., 2016). Individuals who are subjected to ostracism often experience the feeling of being marginalized and ignored by others, which may be attributed to poverty or power disadvantages (Grimalda, 1999). Additionally, social exclusion can either be active, such as in the case of laws that explicitly put a certain group at a disadvantage, or passive, such as when a group’s social exclusion is an unintended by-product of a specific practice or law (Sen, 2000). The term is dynamic, implying that social exclusion exists and that it influences the lives of not only those who are excluded but also of those who are not.

The concept of social exclusion mostly focuses on its consequences on individuals, such as how it affects their self-concept and self-esteem (Gerber & Wheeler, 2009; Richman et al., 2015). Research has also shown that social exclusion compromises one’s sense of belonging (Williams & Nida, 2011) and hampers one’s ability to attain self-fulfillment in all aspects of life (Nussbaum, 2006). In studies on organizations, Robinson et al. (2013) and Yang and Treadway (2018) highlighted workplace ostracism, a practice by which intentional social exclusion prevents employees from performing to their full potential. Other studies on social exclusion also emphasized its consequences, such as an impact on life satisfaction (Arslan, 2019), an increase in negative effects such as meaninglessness and lack of emotions (Twenge et al., 2003), an increased likelihood of unethical behaviors (Kouchaki & Wareham, 2015; Yang & Treadway, 2018), a higher possibility of aggressive tendencies (Poon & Wong, 2019), and even the likelihood of becoming radicalized (Renström et al., 2020).

Although the topic of women and their difficulties in the workplace is not an entirely new line of thought in the field of business ethics (Kenny & Donnelly, 2020; O’Neil et al., 2008; Phipps & Prieto, 2021), little attention has been paid to the underlying context that imposes the social exclusion of women to keep them from occupying positions of power or gaining critical resources. While informal institutions of patriarchal structures are observed in Korea (Byun & Won, 2020; Gress & Paek, 2014; Lee, 2018), the literature offers little evidence on how these informal institutions enact and shape informal networks (Minbaeva et al., 2022). Specifically, the literature is relatively silent on exploring the interrelationship between informal networks and informal institutions to explain gender-based social exclusion in the workplace (Georgiadou & Syed, 2021). Exploring social exclusion through institutionally driven informal networks can, therefore, fundamentally serve as a means to understand how such structures inhibit women’s progress and impede ethical business practices.

### Informal Institutions and Informal Networks

#### Informal Institutions

Institutions are commonly defined as rules and procedures constructed by symbolic elements or social activities that enable or limit an individual’s behavior (North, 1991; Scott, 2008). Formal institutions refer to contracts and regulations that are easy to modify (North, 1991), while informal institutions include customs, traditions, norms, and religion, which are difficult to change (Williamson, 2000). North (1990, p. 201) defined informal institutions as “a set of rules, compliance procedures and moral and ethical behavioral norms designed to constrain the behavior of individuals in the interests of maximizing the wealth or utility of the principals.” They can also be described as “(1) extensions, elaborations and modifications of formal rules, (2) socially sanctioned norms of behavior, (3) internally enforced standards of conduct” (North, 1990, p. 40). In essence, informal institutions are rooted in the social fabric and societal traditions linked to culturally prevalent understandings and social expectations (Horak, 2014), and they are frequently characterized by reciprocity, cooperation, and the exchange of favors.

Informal institutions have been studied in a variety of scholarly domains (Campbell, 2004; Peng et al., 2009; Webb et al., 2009). The role of institutions in general has been documented in organization studies (e.g., Abdelnour et al., 2017), international business (Doh et al., 2017; Estrin & Prevezer, 2011; Kostova & Hult, 2016), entrepreneurship (Gimenez-Jimenez et al., 2020; Harraf et al., 2021), strategy (Yao et al., 2020), and political science (Helmke & Levitsky, 2004). However, scholars have given scant attention to the informal fabric of institutions, particularly in contexts outside of the United States and Western European countries where informal institutions might be strongly embedded due to cultural influences (Cannatelli et al., 2019; Dau et al., 2018). For this reason, Minbaeva et al. (2022) referred to the need to unpack the ‘black box’ of informal institutions to better understand the role of both informal institutions and informal networks.

A common viewpoint in the literature was to consider informal institutions in the context of formal institutions, as if they were two ends of a continuum (see Helmke & Levitsky, 2004; Yao et al., 2020). As conventionally assumed in the literature on institutional voids (Khanna & Palepu, 1997; Kostova & Hult, 2016), Sauerwald and Peng (2013) suggested that “informal institutions gain importance once formal institutions are absent or weak” (p. 854). Doh et al. (2017) further commented that in order to “address shortcomings in formal institutions, they [firms] may rely more heavily on informal institutions” (p. 294). Hence, the view of informal institutions substituting for ineffective or non-existent formal institutions suggests that actors rely on informal structures (Doh et al., 2017; Mair et al., 2012). What this perspective leaves unexplained, however, is the phenomenon of persisting informal institutions when effective formal institutions are in place (Bian, 2022; Horak & Klein, 2016; Minbaeva et al., 2022). By developing a fresh perspective on the dynamics of informal institutions, Minbaeva et al. (2022) explained this in terms of the role informal networks^1^ play and upon which informal institutions rest.

Social practices in Korea are underpinned by Confucianism, which specifies the relationships between parent and child (particularly father–son), husband and wife, ruler and subject, older brother and younger brother, and even between friends. Each of these relationships has certain moral norms, as well as informal institutional guiding principles (see Table 1). For instance, children are expected to show filial piety, just as parents are expected to care for their children. There is also an expectation of order between younger and older people, where the younger individuals are expected to show respect and obedience to the seniors, and the seniors respond with consideration and protection. These interpersonal connections serve as the cornerstone of social institutions because they require each person to act in accordance with their function in order to maintain an ordered and peaceful society (Chen & Chung, 1994; Du, 2015, 2016; Jingjit & Fotaki, 2010; Yao, 2000). Relationships are mainly dominant–subservient in nature, such as the subordination of the lower status to the higher status and the younger/junior to the elder/senior. Thus, social practices that result from these moral norms place a strong emphasis on paternalism, gender disparities, social hierarchies, fidelity, and loyalty (Koh, 2008; Yeh & Xu, 2010).

**Table 1:** Confucian relationship ethics, informal institutions, and social practices

Relationship	Moral norms	Informal institution (general)	Social practice
Ruler-subject	Benevolence	Social hierarchy	Ruler has to show justice, and the subject shows loyalty
Father-son	Filial piety, love between father and son	Paternalism	Paying utmost respect to the eldest male in the organization, family, or community; (eldest) sons are granted family privileges [women play an inferior role]
Husband-wife	Differentiation between man and woman, submissiveness	Gender-inequality	Men are superior to women, women serve men
Older brother-younger brother	Order between younger and older people	Loyalty	Juniors owe seniors respect and obedience, and seniors owe juniors consideration and protection
Friend-friend	Faithfulness, trust among friends	Fidelity	Friends (or ingroup members) collaborate and show mutual faith to each other [non-hierarchical relationship]

*Sources* Chen and Chung (1994), Jingjit and Fotaki (2010), Koh (2008) and Yao (2000)

#### Informal Networks

Informal networks can be defined as culturally embedded relationships that are formed by informal dyadic ties between individual actors (Minbaeva et al., 2022). At the behavioral level, informal networks produce social practices, and at the structural level, they produce social structures through social interactions. For instance, studies that examine transition economies look at formal institutions (e.g., Rodríguez et al., 2022), and an increasing number of studies also focus on informal institutions (e.g., Puffer et al., 2010; Sauerwald & Peng, 2013). These institutional conditions enable particular structures in terms of business connections and how things are done. Informal networks are underpinned by sociocultural norms and traditions, which are firmly embedded within informal institutions (Minbaeva et al., 2022). Strong informal network relations can continue to exist, which can ensure the effectiveness of the institutions on the one hand but also challenge and raise ethical concerns, particularly for individuals who are unable to access such networks.

As informal institutions shape norms and actors’ beliefs, a shared meaning emerges among actors, determining ‘the rules of the game.’ These behaviors and actions become ingrained in social practices and structures; who are connected to whom in an informal network is determined by these practices, which are founded on shared norms and values. Similarly, who can get access to this informal network is also determined by the social practices that become part of that important element of a cultural identity. This is clear in the case of Brazilian *jeitinho* (Lee Park et al., 2018) as well as in the *wasta* context in the Arab World (Zhang et al., 2021). *Wasta*, which is described as “a parallel inegalitarian system that categorizes people according to their connections,” has a dominating and “prevalent masculine nature” (Alsarhan et al., 2021, p. 131). Such social structures and practices dictate social expectations and norms “related to the traditional role of women” (Alsarhan et al., 2021, p. 131). In this way, these social norms become a way of life that is deeply ingrained in society.

In Korea, *yongo* has been described as an affective informal network (Horak & Yang, 2016; Yang & Horak, 2019), and *yongo* is a closed informal network (Horak, 2018). *Yongo* ties are unique compared with those in the West and other East Asian nations (Horak & Taube, 2016; Horak et al., 2019). They are intricately woven with institutional and cultural factors. As a result, these informal networks do not exist in a vacuum; rather, they are profoundly ingrained in Korea’s informal institutions, which explains why *yongo* still persists today (Horak & Klein, 2016). *Yongo* has a substantial impact on management practices in Korea, but these informal networks are still “an implicit and insignificant side issue” in management research (Horak, 2014, p. 82). The next section expands on the informal networks of *yongo* in the Korean setting.

### Yongo

The English translation of *yongo* describes the syllable *yon* as an affective tie and *go* as the preexistence of ties due to a shared background. Thus, *yongo* can be described as consisting of affective ties between individuals that share something in common; they operate as particularistic and rather exclusive informal networks (Horak, 2014; Lee et al., 2022). Koreans consider *yongo*, the informal networks embedded within the society, as an important source of identity construction and emotional bonds or affective ties.

The affective nature of *yongo* is an important aspect of tie and network cohesion. Different from, for instance, the Chinese *guanxi* and Russian *blat/svyazi* networks (Burt & Burzynska, 2017; Chen et al., 2013; Ledeneva, 2003, 2008; Puffer et al., 2010), *yongo* is in principle cause-based, not purpose-based, as *yongo* is traditionally based on three preexisting ties: (1) the same educational institution that actors attended, isochronous or not (in Korean: *hakyon*,학연); (2) blood ties, attained by belonging to the same family (nuclear and extended; *hyulyon*, 혈연); and (3) social ties based on the same place of birth (i.e., hometown; *jiyon*, 지연). In Korea, these ties are immutable and irreversible; they are quasi-predefined and determined by birth. The traditional center of *hakyon*, that is, high school ties, were also fixed because one usually attended high school close to one’s birthplace. Today, *hakyon*-based ties are to a great extent defined by university affiliation. In an extension to the traditional *yongo* networks, other affective ties may extend or complement *yongo* in today’s network relationships, such as informal ties established during military service or through attending informally organized gatherings of former co-workers.

Since it is common in Korean society to distinguish between in-groups and out-groups, *yongo* can be regarded as exclusive, and it determines whether people belong to an in- or out-group. Inside a *yongo* circle, there is “flexibility, tolerance, mutual understanding as well as trust. Outside the boundary, on the contrary, people are treated as ‘non-persons’ and there can be discrimination and even hostility” (Kim, 2000, p. 179). *Yongo* characterizes trustful emotional ties in Korean society, deeply engrained in a Confucian value system (Kim & Bae, 2004; Yee, 2000). Loyalty among its members prevails and is pronounced. On a network level, *yongo* works between members belonging to each respective camp. That is, bridging networks (Burt, 1995) between, for example, members of universities A and B (i.e., *hakyon*-based) is against the behavioral norms of *yongo*, as rivalry and competition can exist between each camp. Bonding within *hakyon*, *hyulyon*, or *jiyon* ties, however, is the norm.

### Yongo in the Workplaces in Korea

Most of the research conducted in the past two decades primarily centered around the question of whether gender-related social exclusion still exists in the Korean workplace and how the Korean workplace differs from others (for an overview, see Patterson & Walcutt, 2014). Against the background of more than 30 years of existing legislation promoting gender equality, as well as with the election of the first female Korean president (Park Geun-hye, 2013–2017), we observed a gradually positive perception in terms of changes and trends toward gender equality in Korean workplaces. However, informal networks and *yongoism*^2^ may act as a counterforce on progress and development (Georgiadou & Syed, 2021).

One salient characteristic of Korean workplaces is the excessively long work hours. According to the most recent OECD data, the annual work time in Korea is 1915 h/worker, the fifth-longest yearly work time in the world, after Mexico, Costa Rica, Colombia, and Chile (OECD, 2022). This is despite the Korean government’s implementation of significant work time reform to lower the total weekly work hours from 68 to 52 h between 2018 and 2021 (OECD, 2020). The long work hours undeniably leave little time for both men and women to devote to family life, and married women in particular have to reconsider regular employment when they start their family or perhaps even postpone parenthood (OECD, 2017, 2019). Indeed, the gender pay gap is more pronounced in Korea than in other countries, particularly for married women who face the motherhood penalty (Kim et al., 2016; Yim, 2018).

The issue of gender inequality and discrimination^3^ represents the professional struggles of Korean female professionals (Cho et al., 2015). In fact, Korea’s three largest companies (in terms of revenue) have a low percentage of women occupying the top leadership positions. Women make up only about 5.4% of the executives at Samsung Electronics Co., a mere 2.5% at Hyundai Motor Co., and 3.6% at SK Holdings Co. (Jeong, 2020). Ham (2021) highlighted that the gender gap in the Korean labor market even widened during the COVID-19 pandemic, with more women than men taking a leave of absence from work. Patterson and Benuyenah (2021) also acknowledged the absence of women in top management positions. The glass-ceiling phenomenon is undoubtedly a real challenge in the Korean labor market, with significant economic and social implications. In this study, we specifically examine how both informal networks and informal institutions play a role in socially excluding women in the workplace. We then explore how MNCs can play an active role in fostering the careers of women professionals by encouraging inclusive leadership and social inclusion.

## Methodology

Given our focus to understand a novel context (i.e., informal networks), we adopted an exploratory research approach to learn about the perceptions, attitudes, and beliefs of the respondents about the topic (Pratt, 2009). We chose in-depth interviewing as our method, providing the interviewees an opportunity to share their beliefs and experiences. This method not only facilitates obtaining purposeful information through in-depth conversation but also enables an analysis of emergent and recurring themes (Eisenhardt & Graebner, 2007).

In-depth interviews were conducted in Seoul and surrounding area, in four interview waves (2009, 2012, 2014, 2019). The time frame of data gathering was due to several factors. First, it should be noted that topics like gender discrimination and *yongo* are considered sensitive and that it may be considered taboo to openly talk about them. In a Confucian environment, gender discrimination is often not seen as such; rather, it is viewed as something that comes naturally. Hence, respondents, particularly local male respondents, were often less than willing to talk about it. Given the topic, it was challenging to find enough participants. Second, eliciting the perspectives of top female Korean leaders also proved difficult because there are so few female business leaders in Korea. Further difficulties arose due to the fact that we had only a short period of time to travel and gather data in Korea while working abroad ourselves. After four field visits over the years, we had gathered enough interviews to answer the research questions and ensure a sufficient level of authenticity, reflexivity (Jonsen et al., 2018), and rigor (Köhler, 2016). The time frame, spanning a 10-year period, was useful for identifying the persistence of *yongo* and/or any change in perspectives over time.

As noted earlier, the subsidiaries of MNCs operating in Korea served as the context of the study. Given Korea’s male-dominated corporate culture, there are not many experienced female managers in upper management positions in Korean firms other than in MNCs. Despite their qualifications, women are often discriminated against when trying to enter the job market or be promoted in their careers. Jeong (2019) reported on this entrenched patriarchal culture, where women are frequently questioned about their plans for marriage and having children, their dating lives, and even whether they would wear more makeup to look more attractive or professional. Conversely, MNCs are purported to be strategic through the implementation of more egalitarian Human Resources (HR) practices, with empirical evidence pointing out a higher proportion of women CEOs in MNCs than in Korean companies (Cho et al., 2019; Siegel et al., 2019). We anticipated that MNC subsidiaries would, therefore, be more proactive in creating an environment that promotes equal opportunities, which is why we focused on MNCs in this study.

## Data Collection

We used the key informant technique (Tremblay, 1989) to select interviewees, using a purposive sampling strategy (Miles & Huberman, 1994). Key informants for our study were chosen based on their expertise and whether/how they could provide insights into workplace activities. We selected those who (1) occupied a relevant job position, (2) were knowledgeable, (3) were able and willing to openly talk about their experiences, (4) were objective, and (5) were impartial on the research topic (Tremblay, 1989). Given the cultural norms in Korea, interviewees preferred talking to a person they already knew or were acquainted with through a third person. We used nonprobability sampling by contacting individuals who had preexisting relationships with one of the researchers through the latter’s prior working and living experiences in Korea or through a mutual acquaintance. This contributed to the establishment of trust and an open interview atmosphere (Ryen, 2001). Interviewees with whom no prior relationship existed (approximately 30% of the sample) were approached with the support of the Korean–German Chamber of Commerce and Industry, a local institution with more than 500 members (as of 2019) in Korea.

Semi-structured interviews were used to explore the research questions, with additional probing questions being asked to ensure that the information was sufficiently clear and comprehensive. To ensure that the questions were appropriate and to better understand any potentially overlooked but significant subthemes, the first three interviews conducted tended to be more exploratory. However, as part of the elicitation technique, the later interviews became more focused over the course of the conversation (Johnson & Weller, 2001). We used taxonomically structured questions as an elicitation technique to increase our understanding of the *yongo* construct and what *yongo* meant to the interviewees. According to Johnson and Weller (2001, p. 501), “taxonomically structured questions are excellent for helping the researcher gain an organized understanding of a new topic.” Some of the questions were: “What Korean terms are used for informal networking and what do they mean?” and “How would you describe informal ties and networks typical in Korea? How do they affect your work?” This allowed the researchers to learn the meanings of local terms and become familiar with local phrases and concepts.

We further employed the critical incident technique (Ostrom & Wilhelmsen, 2012). To do this, we asked our interviewees to recall a specific circumstance. On identification of a critical incident, the interviewee was asked, for instance, “How did you perceive this situation?”, “What were your thoughts?”, “What occurred next?”, and “How did you react?” to gain a deeper insight into the case and to observe the interviewee’s reaction. We also considered a suitable environment for each interview, conducting it in a familiar setting where the interviewee felt most comfortable (Herzog, 2012). This was done because the interview location can influence the quality of a participant's answers (Elwood & Martin, 2000). When we asked the interviewees which interview location they preferred, all interviewees decided on their workplace—either in a meeting room or at the personal desk if privacy could be ensured (i.e., with a closed door). All the interviews were conducted in English and lasted 45 min to 1.5 h. Interviews were recorded with the interviewees’ consent; notes were also taken during each interview and completed immediately after the interview had been conducted.

### Participants

While designing the interview study protocol, we paid attention to counteracting bias and increasing validity (Maxwell, 2005) by considering a diverse subject pool across interviewees and industries. The participants were selected from middle and upper-level management, and they held key responsibilities in their organizations. A total of 33 decision makers and policymakers were interviewed, among whom were CEOs or presidents of MNC subsidiaries, vice presidents, chief operating officers, managing directors, HR managers, and department heads.

The nationalities of the interviewees were Korean, German, Dutch, and British, working in subsidiaries of MNCs in different industries, such as automotive, chemicals, logistics, tourism, insurance, and trade (Table 2). All the Korean interviewees had prior experience working for Korean firms before joining the subsidiary of an MNC. Hence, they were able to compare the workplaces of Korean and multinational organizations. The non-Korean interviewees had all worked in Korea for a significant amount of time, usually a decade or more. All of them had either direct or indirect control over decisions about hiring and promotions and were conscious of gender issues in their workplace.

**Table 2:** Overview—Interview participants

No. (#)	Position	Industry	Enterprise category^b^	Interview date	Interview duration (min.)
01	Manager	Consulting	SF	2019	60
02	President & CEO^a^	Pharmaceuticals	MIC	2019	55
03	Managing Director^a^	Healthcare	SME	2019	53
04	President & CEO	Imaging	LF	2019	65
05	Managing Director	Personal care	LF	2019	43
06	President & CEO^a^	Association	SF	2019	55
07	Vice President	Automotive	SME	2019	48
08	CEO^a^	Service	SF	2014	55
09	Manager	Logistics	LF	2014	60
10	Managing Director^a^	Service	SME	2014	60
11	President and CEO	Imaging	LF	2014	93
12	COO	Insurance	MIC	2014	45
13	Vice President	Automotive	LF	2014	60
14	Manager	Steel	MIC	2014	83
15	President	Chemicals	MIC	2014	46
16	Director	Chemicals	MIC	2014	46
17	Director^a^	Tourism	MIC	2014	55
18	President and CEO	Trading	LF	2014	70
19	Product Manager	Automotive	MIC	2014	64
20	Managing Director	Automotive	MIC	2012	61
21	General Manager	Tourism	MIC	2014	38
22	Manager^a^	Imaging	LF	2014	58
23	Managing Director	Logistics	LF	2009	61
24	COO	Automotive	LF	2012	36
25	President	Consulting	SF	2014	82
26	Manager^a^	Consumer goods	MIC	2014	57
27	President and CEO	Chemicals	MIC	2012	94
28	President	Mech. Engineering	LF	2012	67
29	President and CEO	Imaging	LF	2009	54
30	President and CEO	Automotive	MIC	2009	62
31	President and CEO^a^	Chemicals	MIC	2009	42
32	Vice President	Automotive	LF	2009	38
33	President	Automotive	LF	2009	54

^a^Female

^b^By number of employees worldwide. Classification is according to the Organization for Economic Cooperation and Development (OECD): Small Firm (SF): ≤ 50 employees; Small and Medium Enterprise (SME): ≤ 250 employees; Large-Firms: Enterprises with 30,000 employees and more; Multi-industry Companies (MIC): Large firms that do business in more than two industries

### Data Analysis

We followed the method by Gioia et al. (2013) and Gioia et al. (2022) to analyze the qualitative data. The method emphasizes a carefully determined data structure and theoretical abstraction based on the interviewees’ responses, and it has been used in a number of studies (e.g., Alsarhan et al., 2021; Spieth et al., 2019; Suseno & Abbott, 2021). It allows for a close link between what interviewees say and the abstract themes as part of the analysis (Gioia et al., 2022). Saturation occurred after approximately two thirds of the interviews had been completed and no new codes were identified. Further coding of any new themes was no longer required in this case because there was no substantial new information added (Bowen, 2008).

Specifically, the analysis method includes both a first-order analysis (using informant-centric terminology and codes) and a second-order analysis (using researcher-centric concepts, themes, and aggregate dimensions). This simultaneous reporting of the researcher’s and the informant’s inputs allows for a rigorous analysis of the links between the data and the concepts (Gioia et al., 2013). For instance, our interview participants mentioned military ties, evening networking functions, and the networking of women in related professions. These informant terms were coded as first-order concepts. As the coding continued, we began to notice patterns in an increasing number of initial codes and grouped them into first-order categories based on their similarities and differences. For instance, high school ties, university ties, and military ties were included in the category of antecedents of informal networks.

We then looked for linkages between the first-order code categories to group them into second-order themes. Once this grouping was done, we created the aggregate dimensions that served as the foundation for the emergent framework (Corley & Gioia, 2004). For instance, antecedents of informal networks, gender-based segregation, and homophily were all related to *yongo* networks, whereas women-only networks was relevant to *inmaek* networks (this will be explained in the Findings section). These two themes were then combined to form the aggregate dimension of informal networks. Using the same coding and analysis process, we identified other second-order themes and categorized them as three additional aggregate dimensions (informal institutions, social exclusion, and gender equality policies). In this second-order analysis, we focused on determining whether the emerging themes pointed to concepts that could help us define the phenomenon we were observing (i.e., the dimensions related to the research questions).

**Fig. 1:**
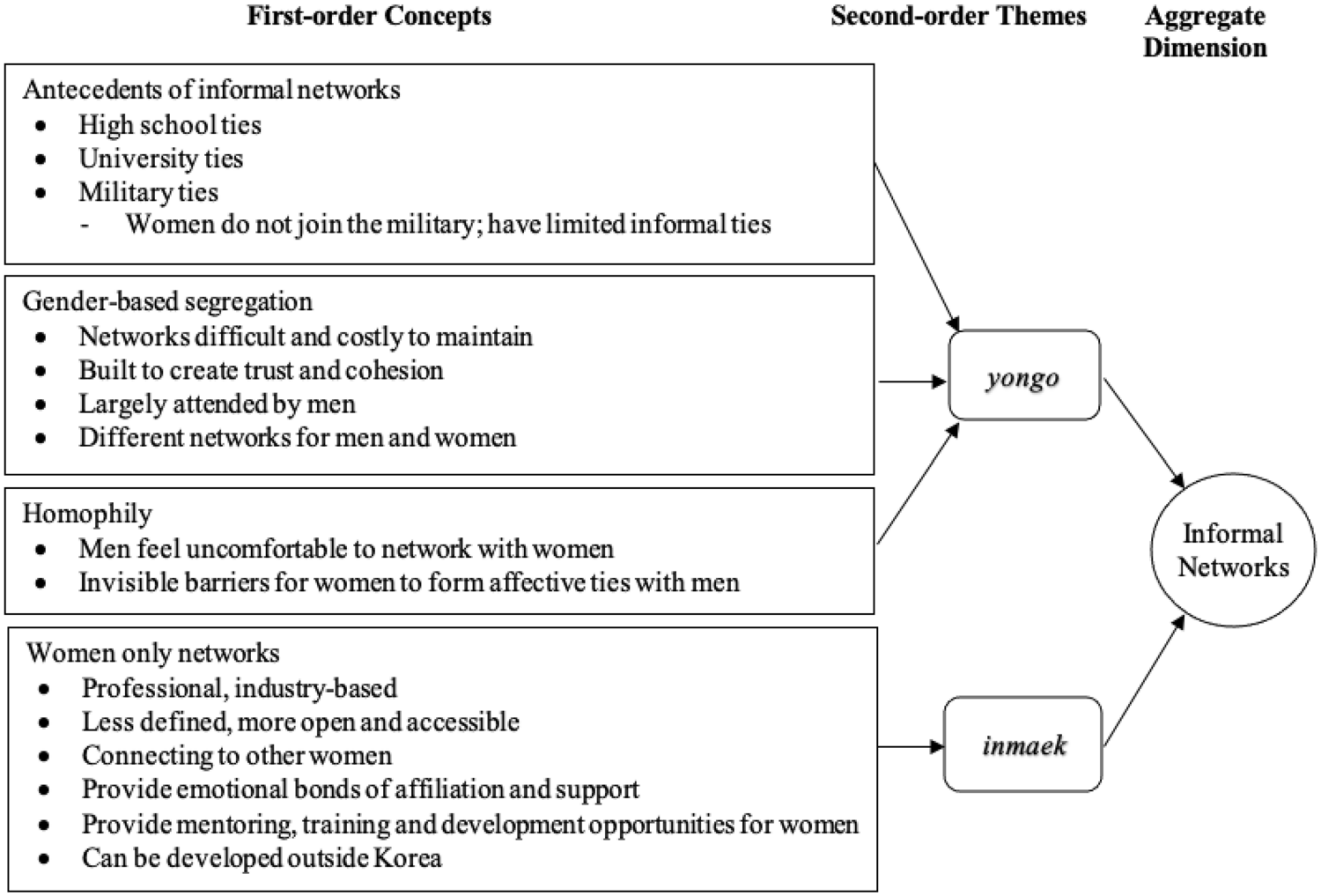
Coding results for the aggregate dimension of informal networks

**Fig. 2:**
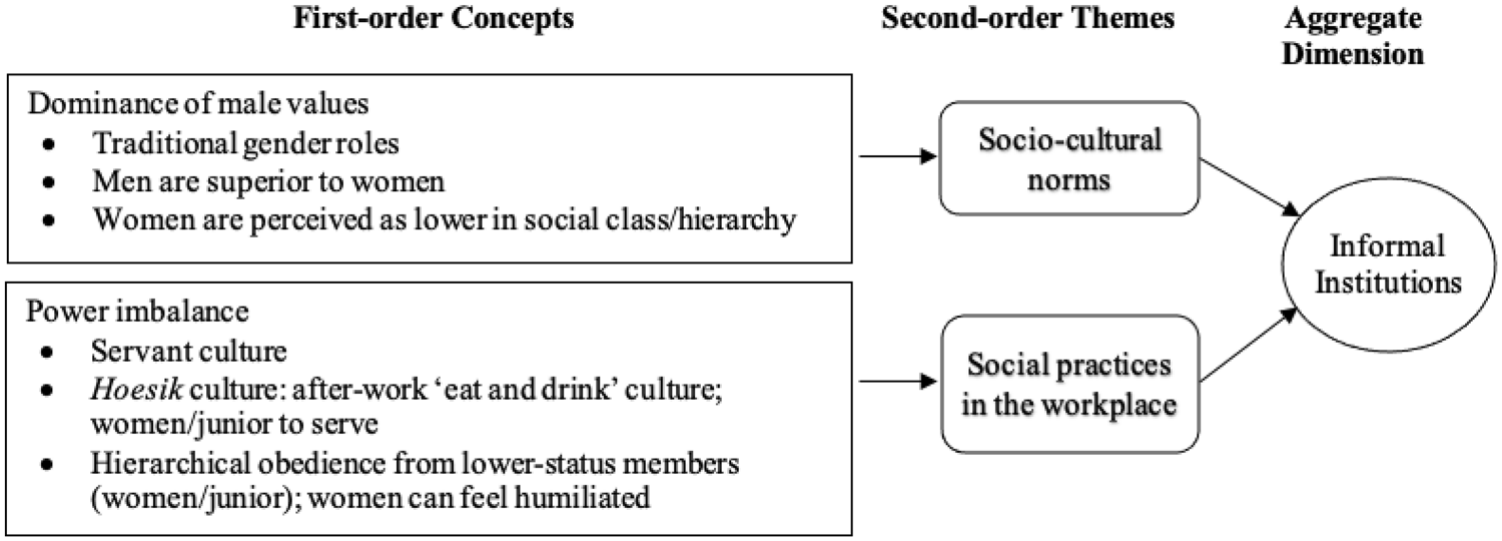
Coding results for the aggregate dimension of informal institutions

**Fig. 3:**
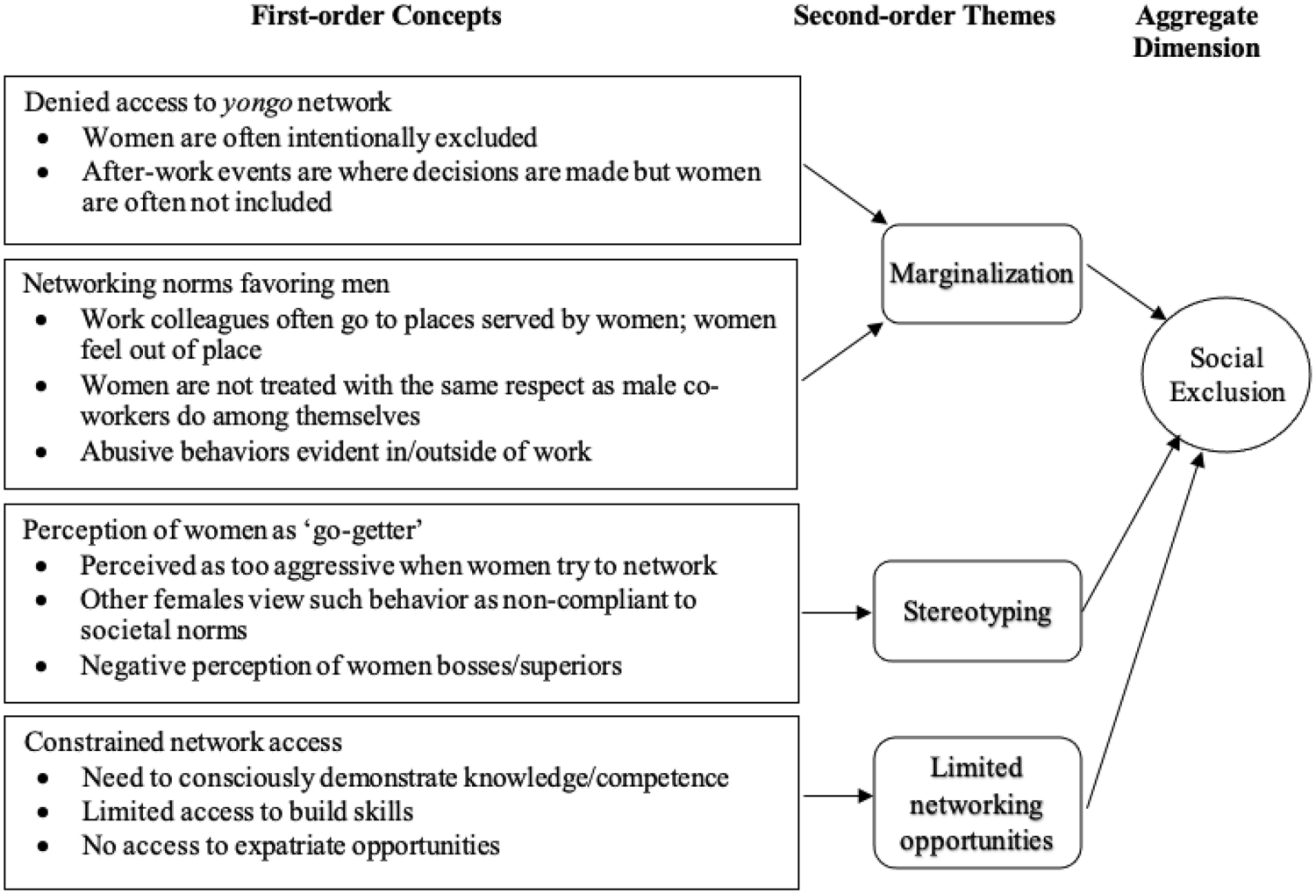
Coding results for the aggregate dimension of social exclusion

**Fig. 4:**
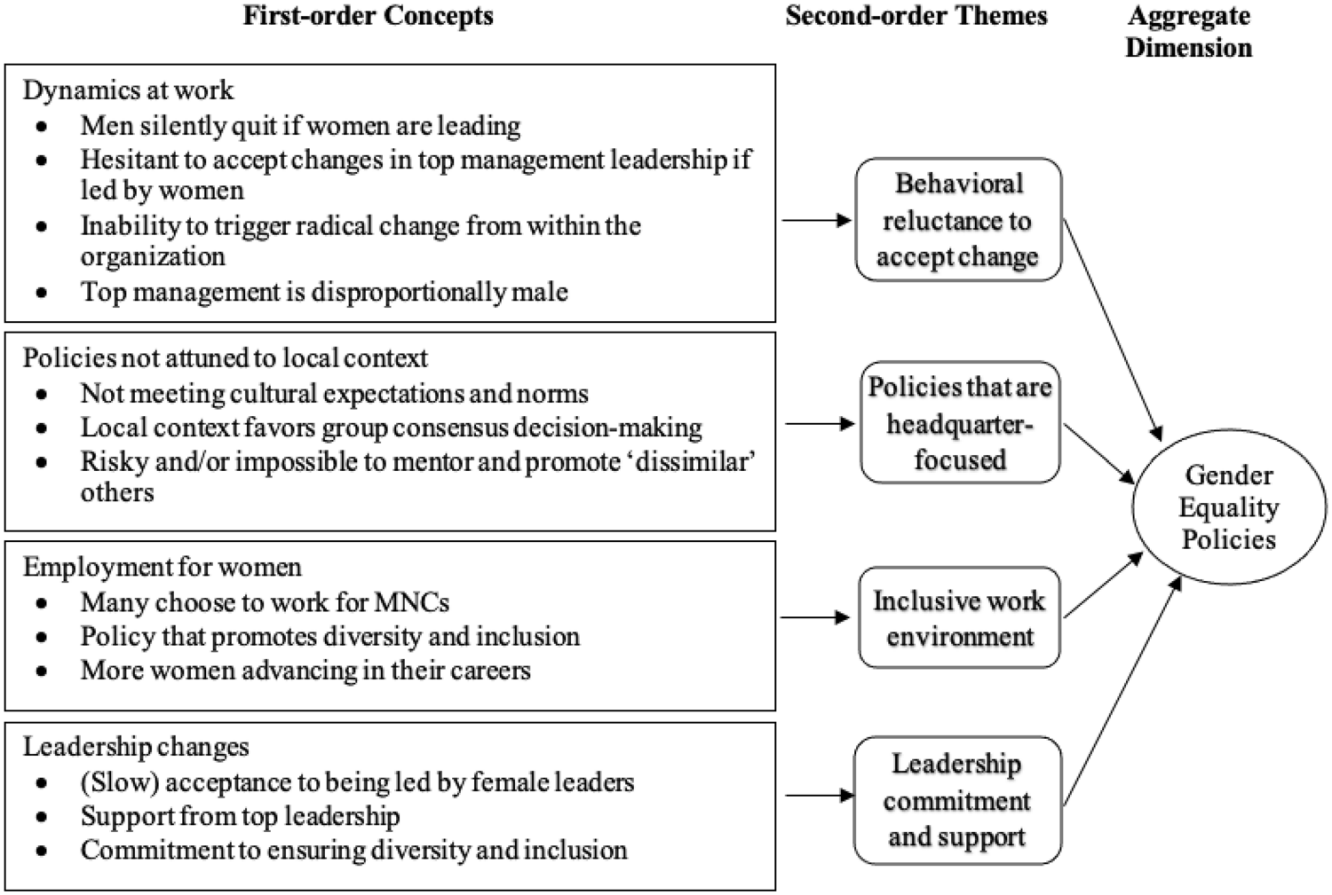
Coding results for the aggregate dimension of gender equality policies

The first-order categories, second-order themes, and aggregate dimensions were then presented in a data structure (Corley & Gioia, 2004). Our overall data structure (see Figs. 1, 2, 3, 4) has 45 first-order concepts that are organized in 14 code categories, which are in turn grouped into 11 s-order themes in our overall data structure. These are further aggregated into four dimensions. This way, the data structure demonstrates not only a system of evidence but also the connections between data and theory.

To ensure external validity, three key informants checked and confirmed the interpretations of the data and final themes. Interrater reliability was assessed by using the test–retest method at different time periods (Mackey & Gass, 2005). Approximately 16% of the randomly selected transcripts were recoded. The interrater reliability was 91%, which is in line with accepted reliability standards (Neuendorf, 2017).

## Findings

### Theme 1. Informal Networks: Male Yongo Versus Female Yongo

Figure 1 summarizes the first-order concepts, second-order themes and the aggregate dimension of informal networks identified in our data. We highlighted the types of informal networks from our data—that of *yongo* and *inmaek*—as the second-order themes.

In describing the informal networks of *yongo*, our interviewees confirmed that *yongo* is principally determined by preexisting ties of *hakyon* (ties based on educational affiliation), *hyulyon* (blood ties), and *jiyon* (ties based on place of birth), although they emphasized the predominantly *hakyon*-based *yongo*. These relationships are largely based on affective ties formed during high school and university years that last a lifetime. Having limited or no preexisting informal networks of *yongo*, particularly *hakyon*-based *yongo*, makes it very difficult to get promoted or to even get a job, as a female manager reported:

When applying for a job it can make a difference when you graduated from a university that has a lot of yongo. I know a case when a female graduate from a smaller university was applying for a job and the recruiters of the firm were asking themselves 'why is she applying for this job, she doesn't know anyone in the firms. She has no yongo in the firm. No one will help her'. (#01)

In addition to the *yongo* developed during university years, our interviewees commented that the norms of forming and building informal ties during military service have a significant impact. Because only Korean men serve in the military, they often form deep and strong bonds with one another. Indeed, the informal ties formed during military service often act as a springboard for the development of even closer ties that lead to business relationships. Given that women are not allowed to serve in military service, they, therefore, have relatively limited *yongo*:

To some extent, it is because men have to go to the army and that is the place where very deep relationships are built. They are as strong as yongo. (#26)

As shown in Fig. 1, our data also suggest that gender-based segregations exist in a variety of economic and social activities. Maintaining *yongo* is time consuming, often costly, and requires efforts that extend beyond the business sphere. With frequent engagement in in-group activities, relationships deepen and become more intimate over time with *yongo*, as trust and cohesion develop on both dyadic and network levels. Even while our interviewees stressed the importance of networking after work to strengthen these informal ties, most of these events are attended by men only. Similarly, others stated that despite the fact that women desire to mix with their male co-workers, their networks are typically limited and confined to their own gender as a result of their inability to break down the invisible barriers that exist between men and women.

I believe for the few businesswomen in Korea it is difficult to join the informal events in the evening. Korean male executives prefer to be among other men, I think. (#08).

Yongo needs to be maintained, for example through dinner and drinking events after work, and that is done on the executive level most often among men only. (#22)

Additionally, we learned from our interviews that there is the aspect of homophily in informal networking in Korea, where men are simply uncomfortable building *yong*o with female managers/colleagues over dinner and drinks after work. *Yongo* cannot be activated and established without the capacity to break down these invisible barriers and develop affective ties with people of the opposite gender. Due to gender-related homophily, which is pervasive not only in the workplace but also in the broader Korean society, women in general are less likely to cultivate *yongo*:

My personal feeling is that women leaders do not want to network among themselves, but they seek to be an accepted member of the male circles. However, when it comes to informal networking for instance for dinners and drinks in the evening it is quite separated. Women meet in women’s groups and men like to stay among themselves. (#22)

We have a diversity policy in place, but yongo, especially in upper management, is quite a male thing. (#26)

Our findings also revealed intriguing patterns of discussion, one of which demonstrates how Korean women professionals network when the networking opportunities with their male colleagues are limited. When we asked our respondents about details on what opportunities female managers have, we discovered that women do network, but in a different way than men typically do this. One interviewee noted this different way of networking for women professionals in Korea:

There are women networks in business, but they constitute in a different way than male networks. (#05)

Our findings suggest that female professionals may not and cannot rely on typical informal networking (*yongo)* in the workplace, as their access and ability to proactively nurture *yongo* are limited. Women use another form of networking that is less defined, more open, and accessible to anyone. We found evidence of this different way of female networking, which is categorized under the theme of *inmaek*. *Inmaek* is comparable to Chinese *guanxi*; it is accessible and describes the social relationships and ties people develop during their life. Several interviewees noted that female managers can develop *inmaek* by participating in professional industry-based women-only networks. It is a promising approach, circumventing male-dominated *yongo* ties for women to establish these networks for their job success and career progression. Women-only networks may provide not only the tangible benefits of being connected to formal and informal networks of other females but also the emotional bonds of affiliation and support that are critical for policy change. Additionally, they provide women the opportunities for mentoring, training, and development. The growth of *inmaek* is highlighted below:

Their networks, as they develop recently in Korea, are often industry or cross-industry-based networks, like the ‘women in finance’ or ‘women in innovation’ networks, where mostly Korean women gather. (#06)

In a striking contrast to *yongo*, *inmaek* is institutionalized in such a way that it transcends borders; hence, it does not focus solely on Korea, as *yongo* does. As such, women can develop global *inmaek* (see also Lee et al., 2022), which can be described as having a network outside of Korea. A female manager explained:

There are also mixed and international women networks, where businesswomen meet other businesswomen from different countries. Further, international women networks can be found at the political level like for instance the women twenty (W20) network that is related to the G20 gathering, in which delegates can bring in their ideas for recognizing women’s needs for policy reforms. (#06)

In summary, our data revealed that *yongo* is limited for women because it is predicated on preexisting ties formed during university or military service. Women also have restricted access to these informal networks of *yongo* as a result of gender-based segregation. Additionally, we identified the notion of homophily in our data, as men often feel uncomfortable building network ties with women in their professional networks, including at gatherings held after work hours. *Yongo* as such is an exclusive network, which makes it rather closed to female managers in Korea. However, when opportunities to connect to *yongo* are minimal, women professionals utilize their *inmaek* ties to network (see also Lee et al., 2022, on using *inmaek* as a strategy for foreign firms in Korea to overcome the liability of foreignness). In a similar vein, women are increasingly learning and utilizing *inmaek* in the workplace to get around obstacles or liabilities associated with being different from their male counterparts.

### Theme 2. Yongo and Informal Institutions

An overview of our findings on *yongo* and informal institutions is shown in Fig. 2. The sociocultural norms and social practices in the workplace served as the foundations for the aggregate dimension of informal networks.

The majority (80%) of our interviewees emphasized the accepted sociocultural norms in Korean culture. One of these is the dominance of male values in the workplace, rooted in Confucian informal institutions. Men are thought to be superior to women, and women are expected to be submissive to men. Confucian sociocultural norms, according to our interviewees, constitute the foundation of expectations of what roles women are supposed to play in the family, at work, and in society (see Table 1 earlier). Thus, women professionals have a hard time accessing and maintaining *yongo* networks as a result of societal perceptions of traditional gender roles governing housework, such as the idea that women should be more involved in domestic responsibilities than men. This is explained by a female HR manager:

Male networks often build around the traditional pillars like military time ties, university, or high school ties, and they are maintained by joint activities like hiking, golfing, dinner and drinks events. Women don’t have the time as they are managing the household and kids after work. (#06)

Women are well aware of the importance of *yongo* in business, yet they are often unable to access and leverage it. Our interviewees noted that the sociocultural norms dictate that informal networks are socially welcoming of men and less so for women. While many view *yongo* as desirable to access because they are very influential in achieving an advantageous outcome, the paradox is that dominant male attitudes and perceptions of women’s role in society take center stage.

Traditionally women do have a limit when it comes to yongo because business is something men do and women usually stay home. (#08)

Another facet of informal institutions, as evidenced by the data, is social practices in the workplace that are represented by power imbalance (see Fig. 2). Our interviewees mentioned that the social practice of servant culture,^4^ in which women serve men or the young serve the elderly in exchange for benevolence, is common in Korean society (see Table 1). This is reflected in the male dominance and the acceptance of men as being superior to women. When they are forced to serve as servants to their seniors or superiors, women who wish to be regarded as equal to their male counterparts can feel humiliated, but they frequently have to put up with such behavior.

One example brought up in our interviews was that of the pervasive and powerful informal institution of after-work gathering etiquette, known in Korean as ‘*hoesik*’ (회식). *Hoesik* refers to an entrenched after-work eat-and-drink culture that, while informal and after-work in nature, adheres to a strict etiquette. Although it is often voluntary, attendance is anticipated, with an assumed expectation for the participants to drink alcohol. Our interviewees also mentioned that there is an expectation of lower-status organization members displaying hierarchical obedience to higher-status members at work. Informal institutions reflecting a Confucianism-influenced value system and societal expectations are supposed to be followed; this includes knowing your place in the social hierarchy. Women, as well as junior employees, are expected to serve men (who are mainly seniors) by pouring them drinks and to obey the social hierarchy determined by seniority (age), tenure in the company, and job title. During *hoesik,* for example, particular behavioral norms are expected to be followed.

When meeting informally in the evening we actually have traditions that are not in favor for women. For example, either the youngest at the table or a woman has to pour the drinks for the most senior male managers. That’s a tradition and that puts people of different age or gender in a lower position. (#22)

In summary, traditional gender roles, which are deeply embedded in daily life, are still very much evident in the workplace. Our interview data support the notion that informal institutions impact how informal networks function and vice versa (Minbaeva et al., 2022). When informal institutions are underpinned by gender segregation and the conventional conception of gender roles, they serve as gatekeepers, that is, they restrict access to informal networks. These informal institutions of sociocultural norms and social practices in the workplace effectively restrict women from building, strengthening, and leveraging informal networks. Additionally, the dominance of male values and the power imbalance informal institutions represent continue to shape the informal networks of *yongo*. As a result, *yongo*-based informal networks and informal institutions are intertwined, and the relationship between them is intricate and deeply rooted.

### Theme 3. Yongo, Informal Institutions, and Social Exclusion

Our findings for this theme about the connection between *yongo*, informal institutions, and social exclusion are summarized in Fig. 3. All our interviewees mentioned how women are socially excluded in the workplace. Our findings revealed that social exclusion in the workplace falls under three categories: marginalization, stereotyping, and limited networking opportunities.

Regarding marginalization, many of our interviewees stated that women are typically denied access to *yongo* networks. Additionally, if they want to network with their male superiors or co-workers, they have little alternative but to embrace prevalent norms and social behaviors. Women have a long history of being socially disadvantaged in society, and as a result, they are often intentionally marginalized in the workplace. It is, therefore, considerably more difficult for women to be a part of *yongo*; they are intentionally denied access to the network that can give them critical information or resources. As one of our interviewees described it:

Women are often purposefully excluded from networking. Sometimes after-work events consists visiting two or three venues. Although we all went together to the first venue, it is expected that the female managers go home after the first venue so that men can be among themselves and proceed without the female managers to the second and third venue. It really excludes female workers from understanding important information, issues, and decisions. (#01)

Several of our interviewees further mentioned that there are ingrained networking norms that favor men, which consequently leads to the marginalization of women at work. Many of the people we spoke with mentioned that decisions are often made at after-work activities. As one female interview participant, a manager in the service industry, noted, it is not uncommon that after *hoesik*, participants go to other places for drinks and/or to *noraebang* (Korean for karaoke bar). However, it is a common observation that male employees often prefer bars where they are only served by women, and that puts their female colleagues in an awkward and uncomfortable position, making them steer clear of these places. Thus, developing strong relational bonds, which is commonly accomplished by networking outside working hours, is an activity women are often excluded from. Within the circle of co-workers, men also do not treat women with the same respect with which male co-workers treat each other. Because of the nature of some of these informal gatherings, women often choose to exclude themselves from building their networks to prevent being humiliated or harassed. Abusive behavior toward women does happen, both in and outside of the workplace:

I went to hoesik with other male co-workers and when we went to the second place it was a noraebang place. So they were drinking and singing and starting to dance together and then inappropriate behavior happened. Many of my female friends experienced the same situation. I think men do not see female coworker as equals. They feel entitled to do that. No one is surprised by this behavior and there are unfortunately no company rules in place to ostracize this behavior. (#01)

The second category in relation to the social exclusion of women in the workplace is the stereotyping of women. Our interviewees noted that Korean women are generally hesitant to network, particularly at the upper level. This stereotyping of female professionals and their hesitation to network exacerbate the issue of social exclusion. Networking outside of work could easily be misconstrued—women professionals can be stereotyped as go-getters, hoping to gain an advantage by networking with male colleagues. Women can be seen and perceived as trying to aggressively develop their networks without belonging to a *yongo* camp. Other women may not be tolerant of this behavior because it deviates from Korean female behavioral norms. At the same time, men may be reticent to include women in their networks because of the stereotype of female go-getters. This is particularly evident at the upper management level. While many women are conscious of their careers, they are hesitant to network informally, which further inhibits their access to resources, opportunities, and career progression. Men, on the other hand, may find it easier to network with other men and nurture their *yongo* within and outside of the organization.

I think that on lower management or staff level men and women mix more as it was in the past. They go out for socializing more and more. But at the top management level, one sees this rarely. (#13)

Associated with the category of stereotyping, our interviewees also discussed the perception of women bosses/superiors. Several interviewees highlighted that changes are slowly taking place in Korean workplaces, but there are deeply ingrained perceptions of women bosses/superiors, which may further serve as a force of social exclusion for them. The following quotes reflect this persistent view:

Over the past couple of years, I feel that there is no open talk anymore where people openly say they don’t want a female boss or say women should better stay at home. But I’m sure the opinion has not really changed much overall. (#04)

I have the feeling that right now we see a significant cultural change in Korea in form of younger women managers entering corporate boards. That seems to be a trend, I assume driven by the new female President. Whether this is a long-term trend, that I hope, or not remains to be seen. A problem might be that there are just not many women managers available for such a position and I am quite sure that Korean firms will not hire non-Korean female board members as companies in the US or Germany are doing. However, I believe the Korean society is in a transformation process. (#24)

The third category we found in our data that is related to the social exclusion of women is the limited networking opportunities for women. Women frequently feel compelled to demonstrate their knowledge and competence and, to some degree, the desire to want to be involved in informal networking activities with male superiors and colleagues. Many have a hard time breaking down the barriers to network with their male colleagues.

Women’s inability to activate and cultivate *yongo*, combined with strong informal institutions that translate into societal expectations regarding the role of women,^5^ often result in limited career opportunities for women, especially when it comes to acquiring today’s important skill of international experience.

In the past women were excluded from certain career paths. Expatriate positions abroad, for instance, were often solely filled with male candidates. When pregnant it was quite common to leave a firm and stay at home. Today there are more opportunities for women but change is happening very slowly, and acceptance by society is still rather low. Women in middle management positions are most affected. They carry a double burden by trying to manage their careers in addition to organizing the household and childcare. In most families, expectations of gender roles follow quite traditional conceptions. (#06)

### Theme 4. Implementation of Gender Equality Policies in MNCs

Figure 4 presents a summary of our findings for the aggregate dimension of gender equality policies. Slow but positive changes have recently taken place in Korea, notwithstanding the prevalent belief that men still control businesses. This is in line with indications of changes following the passage of national legislation and the election of Korea’s first female president, Park Geun-hye, an occasion that spurred hopes for further change (Patterson & Walcutt, 2014; Patterson et al., 2013). We heard about women advancing in their careers, particularly during the last two interview waves that we conducted (2014, 2019). Nonetheless, the majority of those women are employed by MNCs. Typically, they gained professional experience in Korean firms before leaving to pursue a senior position in an MNC.

The existence of corporate policies that support diversity and inclusion is one of the main reasons why many graduates choose to work for MNCs, as mentioned by a number of our interviewees. A female manager commented on this:

Foreign firms in Korea have the reputation of being very much in favor of promoting women. In foreign companies, women can make a career. (#22)

We further found indications of changes that are gradually taking place in MNCs in Korea, notably in the area of leadership. One European firm appears to be a role model for how internal barriers can be removed to promote more women leaders. In this case, a high-ranked manager of the subsidiary (an expatriate from Europe) trained and developed a female leader and defended his decision in favor of her against initial concerns from many of the male local managers. This case demonstrates the need to accept change at the higher level of the hierarchy—a change that is about inclusive leadership. Leadership commitment is also required to build an inclusive work environment in which women can be promoted despite objections from male managers, colleagues, and even clients. Future female leaders who are capable must be supported and developed, as explained below:

I wanted to promote a woman to be the manager of our service department. I faced massive objections from male colleagues. They believed a woman cannot and should not lead the department, as our customers may not accept that. So, I gave her step by step more responsibilities, such as a coordinator and later project manager and finally I promoted her later. Meanwhile, she is widely accepted and established. Based on these experiences I promoted another woman in our sales department. She is doing such a good job that she is going to receive a sales award this year. By the way, she is the only woman in the sales department; all others are men. However, promoting women requires persistence and a step-by-step approach. Direct promotion is risky as the rejection level by men is quite high. (#11)

However, we also discovered behavioral reluctance to accept change when women are given leadership roles. One of these concerns the dynamics at work when a division/department is led by a woman. It is interesting to note that while our interviewees acknowledged that men might see a woman leading the division/department as progress, it also made them uncomfortable. This is because the ingrained sociocultural norms of informal institutions are somewhat violated. When this happens, men do not try to renegotiate decisions (such as by promoting women); many silently exclude themselves from the situation, such as by quitting their position. That behavior might be an indication of a general awareness of the privileged status of men in Korean society but also of an inability to trigger radical change from within. This is certainly unique in the case of Korean workplaces, as illustrated by the president and CEO of a firm in the imaging industry:

It is interesting to see how the structure in a division changes once you hire a female leader. Over the years men moved out and more positions are filled with females. That could be a sign that men are uncomfortable working for female bosses and chose to leave their position. However, this happens silently, there is no formal escalation. (#04)

The data also revealed that, despite the majority of our interviewees indicating that modest changes had recently taken place, top management in Korean businesses, including MNCs operating in Korea, is still predominantly made up of men, as reflected below:

We actually have only one female leader among 20 other leaders. That’s not much, but in Korea, it’s currently typical. (#22)

When asked about promoting women to top positions in Korean firms, many of our interviewees expressed skepticism. This is because Korean managers rely on group consensus when making decisions and are unlikely to take the risk to mentor and promote a candidate who is not similar to the rest of the top management circle. This exemplifies the behavioral reluctance to accept change (see Fig. 4), with women having limited access to wider networks and opportunities because the top circle is made up of homogenous elite Korean male networks that are seeking consensus, making the implementation of diversity and inclusion policy more challenging. As a result, organizations need to implement policies that gradually lessen this behavioral reluctance to give opportunities to women and underrepresented groups.

The huge Korean business conglomerates are male societies. In Confucianism, the father is the head of the family. In Korean business, it just works the same way. Sure, these days you read a lot about promoting more women in business; some bigger firms have a diversity policy in place, but at the end of the day this changes very slowly. (#33)

The final second-order theme that emerges from our data is the fact that HR policies in MNC subsidiaries are often developed at their (typically Western) headquarters. This can lead to situations in which the policies are not implemented as intended or are misunderstood due to the different cultural contexts in which they were designed. A manager in charge of a firm’s diversity policy mentioned the following:

I think as long as policies are decided in a foreign headquarter, it’s just difficult to make people here understand what they really mean. Korean traditions drive 90% of the decisions made. You cannot change that by policies that are invented abroad. (#12)

When probed further about women’s career progression, our interviewees reported that the local implementation of headquarters’ policies for diversity and inclusion was not entirely effective because of the influence of informal networks and informal institutions, as previously discussed. Local management frequently struggled to satisfy promotion quotas, since there were not enough women managers available for higher-level positions, as highlighted below:

We have a global diversity policy in place since a couple of years ago in order to promote female managers. I take a look at last year’s promotions and saw 80% were male. I asked my manager why this is the case because our target is 50% and he simply said ‘What do you mean? We just don’t have more qualified women here’.” (#12)

In summary, our interviewees commented that while modest changes are occurring at work and some MNCs are establishing more favorable policies for fostering diversity and inclusion, there is still behavioral reluctance to achieving progress and fairness for women.

Additionally, because local informal institutions and local networks are not taken into account, the implementation of policies to promote gender equality for career progression is not yet as effective as it should be in MNCs. Another reason for the slow progress is that there are so few Korean women in managerial roles. Thus, it is becoming increasingly important to design and implement diversity and inclusion policies that include women from the bottom of the hierarchy to the top, while also being proactive in implementing changes across the board that take into account informal institutions and informal networks in the local context.

### Organizing Figures—Summary of Key Themes

**Fig. 5:**
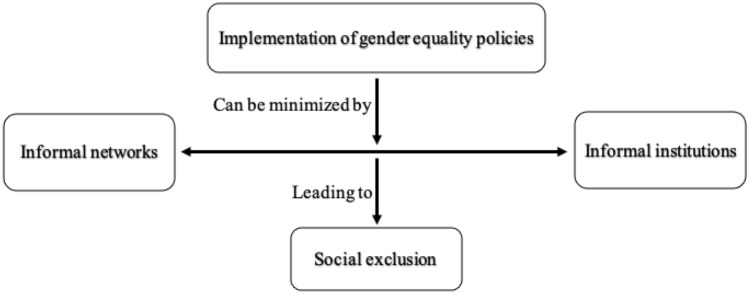
Organizing figures

Following Pratt’s (2009, p. 860) approach to “think[ing] about using organizing figures” in qualitative research, we organized our data and analysis using organizing figures to not only describe the themes but also how those themes fit together. Figure 5 presents a summary of the key themes from our data analysis. Our study highlighted the relationship between the main themes (or aggregate dimensions) of informal networks, informal institutions, social exclusion, and the need to implement gender equality policies in companies. As they are intricately linked, it is clear that informal network access and the role informal institutions play in this regard cannot be considered separately (Minbaeva et al., 2022). The informal networks of *yongo* (Fig. 1) and informal institutions of sociocultural norms and social practices in the workplace (Fig. 2) influence the social exclusion of women (Fig. 3). The ethical challenge of the social exclusion of women in the workplace can only be addressed through the implementation of gender equality policies in organizations, particularly in MNCs to serve as corporate role models for the elimination of gender discrimination and the support of gender equality (Fig. 4).

Under the aggregate dimension of informal networks, the two subthemes of *yongo* and *inmaek* were evident from the interviews. Our analysis also clearly showed the subthemes of sociocultural norms and social practices in the workplace under the aggregate dimension of informal institutions. For the aggregate dimension of social exclusion, our findings indicate concerns with marginalization, stereotyping, and limited networking opportunities for women. Our findings further indicate key actions for implementing gender equality policies in MNCs, such as ensuring that these policies take into account the local context, ascertaining that the highest levels of leadership are open to change while providing development and support for women professionals, and stressing the importance of reducing behavioral reluctance to accept change in the workplace.

## Discussion

So far, few studies examined the social exclusion of women in the workplace (Georgiadou & Syed, 2021; Greguletz et al., 2019; Ozkazanc-Pan ad Clark Muntean 2018) through the lens of informal networks and informal institutions (Horak et al., 2020; Minbaeva et al., 2022). This study sought to address this significant ethical concern of social exclusion of women at work, using Korea as an example and with data gathered from subsidiaries of MNCs. Our arguments are substantially underpinned by the observation that persistent and powerful informal institutions in Korea place women professionals at a disadvantage when it comes to building, cultivating, and leveraging informal networks. Research on this topic is critical because, despite the global efforts to advance gender equality and women emancipation, social exclusion of women in the workplace persists (Georgiadou & Syed, 2021).

This research contributes to the theoretical advancement of the relevance of informal networks and informal institutions by highlighting the embeddedness of informal networks (i.e., *yongo*) in informal institutions (Horak & Yang, 2019; Lee et al., 2022; Minbaeva et al., 2022). Existing literature on the relationship between informal networks and informal institutions is limited (Horak & Yang, 2016; Lee et al., 2022), with Minbaeva et al. (2022) emphasizing the importance of examining how informal institutions contribute to the prevalence of informal networks and the need for this to be empirically examined. The insights from examining the influence of informal networks contribute to an improved understanding of why institutional change is difficult to translate to intended outcomes. Our findings demonstrate how recurring social behaviors contribute to the formation of sociocultural norms (Henry et al., 2004). Due to those norms, it is also challenging to change informal networks. As such, both informal networks and informal institutions are intertwined; they work together to either promote or impede progress.

In the context of Korea, our findings highlight how the informal *yongo* networks are created, nurtured, and leveraged; these *yongo* informal networks are “exclusive and closed to outsiders” (Horak & Yang, 2016, p. 1040). This work further shows that the informal networks of *yongo* are linked and intertwined with Korea’s informal institutions—the country’s sociocultural norms and acceptable social practices in the workplace. In fact, certain societies hold rigid stereotypical expectations of gender roles, with men having traditionally occupied the highest positions in business hierarchies. *Yongo* ties, gender-based segregation, and homophily are rooted in male-dominated sociocultural norms and the perception of traditional gender roles in terms of household chores and childcare. There is also a clear power imbalance in social practices at work, where individuals (e.g., women) desiring access to informal networks must know their place and perform the duties that are expected of them based on both their societal and hierarchical status and their position. Thus, the intricate interplay between the informal networks of *yongo* and the informal institutions acts as an enabler when it comes to the deliberate exclusion of women (from being hired or promoted into higher managerial positions), denying them participation and influence (Georgiadou & Syed, 2021). Our findings clearly support Minbaeva et al.’s (2022) argument that informal institutions play a role in not only enacting informal networks, that is, *yongo,* but also in engendering favoritism for similar others, which can subsequently promote the exclusion of women from opportunities for progress (Horak et al., 2020).

This study also contributes to the literature of business ethics, emphasizing the importance of gender diversity and inclusion around the world (Phipps & Prieto, 2021; Saeed et al., 2021). Even in a nation like Korea, which has long fought for and put legislation in place in support of gender equality, harmony, and personal relationships (Horak et al., 2020; Yeh & Xu, 2010), we found that informal barriers in the workplace remain difficult to overcome. Our findings highlight the ways in which the social exclusion of women manifests in the workplace, with the evidence identifying marginalization, stereotyping, and limited networking opportunities for women. Among other things, both informal networks and ingrained informal institutions make it difficult for women to lead and command men in a business context and typically put women in inferior positions, including at social gatherings after work (*hoesik*). Additionally, we discovered indications of homophily, with men frequently feeling uneasy when they form close personal ties with women (Yee, 2000). All these factors combined produce social structures and practices that leave women with less *yongo*, excluding them from opportunities and power in the world of business.

Our third contribution relates to the broadening of our knowledge on gender equality initiatives and policies at MNC subsidiaries (e.g., Cho et al., 2019; Festing et al., 2015) and the outcomes of these policies in the context of the host country. The corporate culture of MNCs can compensate for the absence or lack of local informal networking, as MNCs provide a more supportive environment for women in the workplace (Siegel et al., 2019). In particular, foreign leaders can make a difference in supporting women’s careers because they are not reliant on or part of the existing informal networks, such as *yongo* ties. In fact, we saw examples of inclusive leadership commitment and support to promote diversity and inclusion, but the effectiveness of diversity and inclusion policies in the host country context was often weak, as these policies were designed in a different institutionalized setting (Cho et al., 2019; Saka-Helmhout et al., 2016). It is evident from our findings that while there is progress being made in Korea, MNCs still have a long way to go to implement their global diversity and inclusion policies. As a result, MNCs must be proactive in considering how gender equality policies may be built and leveraged in relation to the informal networks in the workplace.

Another interesting finding from our data is the presence and application of an alternate networking strategy, *inmaek*, for female managers in Korea. Given that it is more open and less exclusive than *yongo*, *inmaek* has the potential to be further developed and leveraged on the national and international level. In today’s global business environment, in which the norms of social inclusiveness, equality, and meritocracy are desired over male favoritism, *inmaek* may be the more acceptable networking strategy. There is evidence that it is advantageous for women professionals to support other women (Greguletz et al., 2019; Suseno & Abbott, 2021), but most importantly, MNCs can help facilitate the development of *inmaek* by establishing connections among women professionals in their subsidiaries, headquarters, and any other locations where they conduct business. In this way, informal networks are not restricted to the local setting, where it is evident that there are powerful local networks that are difficult for women to access and leverage.

### Practical Implications

Organizations are becoming increasingly aware of the significance of gender equality. Despite progress in legislation and proactive actions by organizations, including MNCs, gender inequality, particularly the deeply rooted practice of socially excluding women, is still an issue in many workplaces. Large listed Korean companies are now required by new legislation to have women on their boards of directors (Bae, 2021). This is certainly a promising start; however, based on our findings and analysis, changes are only slowly happening in organizations. In the context of Korea, women can become resigned to their circumstances and go with the accepted sociocultural norms and social practices; if they want to climb up the corporate ladder, they may have to spend more time gathering with their largely male colleagues at after-work occasions. This raises an ethical concern for organizations; they can no longer simply rely on the normal practices, as it is difficult to break the circle of informal networks and informal institutions.

There are three major implications from the findings of this study. First, in relation to policies, MNCs can set the bar by proactively developing initiatives for the advancement of female managers by ensuring that top management shows commitment and support for such changes to happen, even to the point where, if the decision is not widely accepted by the group (due to homophily and dominant group decision making), this should not be the reason to stop such initiatives. Cross et al. (2002), for example, highlighted that people can fit into informal networks as a matter of intentional behaviors. It is up to the top management team to communicate with the employees and uncover the biases that might prevail in the workplace. They need to educate staff about the gender bias that not only exists in the workplace but is also reflected in the sociocultural norms embedded in cultural traditions.

The second implication is that MNC leaders can promote inclusive leadership and a climate in which informal institutions like *hoesik* culture are not celebrated. This may be achieved by creating a leadership development program that includes awareness training and education on social exclusion and equality. Additionally, MNCs can increase their investments in diversity, equity, and inclusion (DEI) in host countries in a variety of ways, such as hiring, offering opportunities for advancement, diversity training, mentoring programs, and building and promoting DEI roles. It is also necessary to change the workplace culture so that supervisors and employees can openly discuss performance and results without discriminating against women. By actively hiring, promoting, and supporting women, MNCs can achieve higher levels of profitability, and they can serve as role models by doing so (Siegel et al., 2019).

Another practical implication is related to the ways women professionals can build their own informal networks. Our findings reveal the growing importance of *inmaek* and that it should be nurtured at both the organizational and personal level. Women must overcome their own reluctance to use their social ties (Greguletz et al., 2019) by stepping up and leaning in to support one another (Phipps & Prieto, 2021). They also need to become more assertive in challenging the gender structure (Kenny & Donnelly, 2020) and in becoming role models for other women (Suseno & Abbott, 2021). The use of *inmaek* may help women professionals to do so. Additionally, the creation of professional women-only clubs to support the development of *inmaek* can offer an alternative way to develop networks and reduce reliance on the existing informal networks, which were shaped by societal norms, that is, informal institutions, that put women at a disadvantage. The growth of *inmaek* networks was clearly indicated in our interviews, with several respondents highlighting affiliation and support in the forms of mentoring, training, and development opportunities for women. These *inmaek* networks can be developed within a firm by fostering an environment for the leadership development of female employees or even at the broader institutional level to make progressive changes at the macro level.

### Limitations and Future Research

We used the context of MNC subsidiaries in Korea, and this setting ensured that our Korean respondents felt comfortable to openly discuss social exclusion in Korean workplaces. However, we did not have the opportunity to compare our findings with data gathered in Korean firms or *chaebol*.^6^ It would certainly be useful to compare data to identify potential biases, even if access to such data is challenging due to Korean corporate rules that forbid talking to outsiders on such sensitive topics, not to mention the comfort level of the interviewees. Future research can also investigate gender-related social exclusion from the perspective of small- and medium-sized business owners to assess how they perceive *yongo* and the social exclusion of women in their workplaces.

Future research can also examine the similarities and differences between informal networks in different countries, such as *guanxi* in China, *blat/ svyazi* in Russia, *sifarish* in Pakistan, or *wasta* in the Middle East (Nadeem & Kayani, 2019; Zhang et al., 2021), women’s informal networks, and social exclusion in different institutional contexts. Such cross-cultural comparisons could provide an analytical framework for identifying how to utilize informal networks to facilitate certain behaviors and interactions in the workplace. Previous research showed that the informal networks of *guanxi* and *blat/svyazi* tend to integrate women in the workplace (e.g., Burt & Burzynska, 2017; Chen et al., 2013; Ledeneva, 2003), while our study found that *yongo* has the opposite effect. Thus, we recommend further research on social exclusion in relation to different types of informal networks and informal institutions to help design more effective approaches for diversity and inclusion.

Finally, we do not claim generalizability of the findings across settings, as the findings are specific to the context. Our findings should, however, be interpreted as foundational, laying the groundwork for further research on the intersection between informal networks, informal institutions, and social exclusion. Given the paucity of previous research on this topic, this study is significant for its theoretical and practical implications in understanding the existence of informal networks and informal institutions that can foster social exclusion at work. Understanding this context provides a starting point for scholars and practitioners to explore potential courses of action to advance gender equality and promote inclusion and social integration on a global scale.

## Footnotes

1 Following Minbaeva et al. (2022), we use the term *informal networks* rather than *social networks* to refer to ties and networks known under the term *wasta* (Arab World), *blat/svyazi* (Russia and other post-Soviet Union countries) or *yongo* (Korea). By doing so, we emphasize the tensions associated with informal networks, such as its exclusiveness, particularism, favoritism, and moral ambivalence (see Horak et al., 2020).

2 *Yongo* is referred to here as informal networks in the Korean context. When referring to *yongo* behavioral values and norms, we use the Korean term *yongoism* (연고주의).

3 Various recent reports and scholarly works noted that gender discrimination is prevalent in Korean workplaces (Kim et al., 2016; The Economist, 2019, 2021). This is manifested in lower pay for women and a preference for hiring and promoting male employees (Jeong, 2019; Kang & Rowley, 2005; Kim, 2019). In a report by Jeong (2020), a comment from a female employee noted that “[t]he discrimination and depreciation women feel in the workplace is so natural, like air.”

4 Servant culture is often perceived to have its roots in Confucian informal institutions and the inequality and social hierarchy it purports.

5 The process of how social practices are produced as the result of informal networks and embedded informal institutions was described by Minbaeva et al. (2022).

6 The Korean term *chaebol* refers to large Korean industry conglomerates, such as Hyundai, LG, and Samsung.
